# Antifungal and Antiaflatoxinogenic Effects of *Cymbopogon citratus*, *Cymbopogon nardus*, and *Cymbopogon schoenanthus* Essential Oils Alone and in Combination

**DOI:** 10.3390/jof8020117

**Published:** 2022-01-26

**Authors:** Ignace Sawadogo, Adama Paré, Donatien Kaboré, Didier Montet, Noël Durand, Jalloul Bouajila, Elisabeth P. Zida, Hagrétou Sawadogo-Lingani, Philippe Augustin Nikiéma, Roger Honorat Charles Nebié, Imaël Henri Nestor Bassolé

**Affiliations:** 1 UFR-SVT, Université Joseph Ki-Zerbo, Ouagadougou 03 BP 7021, Burkina Faso; sawaigna@yahoo.fr (I.S.); pada235@yahoo.fr (A.P.); pnikiema@gmail.com (P.A.N.); 2 Institut de Recherche en Sciences Appliquées et Technologies, Ouagadougou 03 BP 7047, Burkina Faso; kaboredonatien74@yahoo.fr (D.K.); hagretou@yahoo.fr (H.S.-L.); neroch@hotmail.com (R.H.C.N.); 3 UMR 95 QualiSud, Université de Montpellier, CIRAD, Montpellier SupAgro, Université d’Avignon et des Pays de Vaucluse, Université de la Réunion, CEDEX 5, 34095 Montpellier, France; didier.montet@cirad.fr (D.M.); noel.durand@cirad.fr (N.D.); 4 Laboratoire de Génie Chimique, Université de Toulouse, CNRS, INPT, UPS, 31062 Toulouse, France; jalloul.bouajila@univ-tlse3.fr; 5 Laboratoire de Phytopathologie, Institut de l’Environnement et de Recherches Agricoles (INERA), Ouagadougou 04 BP 8645, Burkina Faso; zidap.elisabeth@yahoo.fr

**Keywords:** *Aspergillus parasiticus*, *Aspergillus flavus*, essential oils, combination, antifungal, antiaflatoxinogenic effect

## Abstract

The antifungal and antiaflatoxinogenic activities of the essential oils (EOs) from the leaves of *Cymbopogon schoenanthus*, *Cymbopogon citratus*, *Cymbopogon nardus*, and their pair combinations were investigated. Antifungal susceptibility and the efficacy of paired combinations of EOs were assessed using agar microdilution and checkerboard methods, respectively. Identification and quantification of chemical components of the EOs were carried out by gas chromatography-mass spectrometry and gas chromatography-flame ionization detector (GC-MS and GC-FID), respectively. Aflatoxins were separated and identified by High-Performance Liquid Chromatography (HPLC) and then quantified by spectrofluorescence. The EO of *C. nardus* exhibited the highest inhibitory activity against *Aspergillus flavus* and *Aspergillus parasiticus*. The combination of *C. citratus* and *C. nardus* and that of *C. nardus* and *C. schoenanthus* exhibited a synergistic effect against *Aspergillus flavus* and *Aspergillus*, respectively. Both *C. citratus* and *C. schoenanthus* EOs totally inhibited the synthesis of aflatoxin B1 at 1 µL/mL. *C. citratus* blocked the production of aflatoxins B2 and G2 at 0.5 µL/mL. Both *C. citratus* and *C. schoenanthus* totally hampered the production of the aflatoxin G1 at 0.75 µL/mL. The combination of *C. citratus* and *C. schoenanthus* completely inhibited the production of the four aflatoxins. The study shows that the combinations can be used to improve their antifungal and antiaflatoxinogenic activities.

## 1. Introduction

Mycotoxins are toxic secondary metabolites produced by various filamentous fungi, mainly belonging to Fusarium, Aspergillus, Penicillium, and genera [1]. They contaminate food, feed, and various agricultural commodities either before harvest or under post-harvest conditions [1,2]. Some of them have been demonstrated to have disease-causing activities, including carcinogenicity, immune toxicity, teratogenicity, neurotoxicity, nephrotoxicity, and hepatotoxicity [3]. Currently, more than 300 mycotoxins are known and possess wide variations in fungal origin, structure, function, and biological effect but only a few of them appear to have a significant effect on health and agriculture. Among them, aflatoxin- (B1, B2, G1, and G2) producing species are considered as the most important in terms of prevalence, toxicity, and impact on human and animal health [4,5]. The International Agency for Research on Cancer (IARC) classed aflatoxins as carcinogenic (Group 1), potentially carcinogenic to humans [6,7,8]. The cereals and oilseeds are the most contaminated by the mycotoxins produced by *Aspergillus flavus* and *Aspergillus parasiticus* [9]. The environmental, agricultural, and food storage conditions in Africa favor the development of molds of the genus Aspergillus and the production of aflatoxins [10].

Several approaches have been proposed to minimize mycotoxin contamination in food [3,11]. For a long time, chemical compounds have been used to prevent fungal growth and mycotoxins contamination [12,13]. However, the indiscriminate use of chemical fungicides has important drawbacks, including effects on human and animal health and the environment, and an increased number of resistant isolates [14,15,16]. 

EOs of aromatic plants could potentially serve as effective alternatives to synthetic chemicals for the control of food contamination by Aspergillus spp, because of their relatively low toxicity and their biodegradability [17,18]. The objective of the present work was to investigate antifungal and antiaflatoxinogenic effects of EO alone and in pair combination of *Cymbopogon citratus*, *Cymbopogon nardus*, and *Cymbopogon schoenanthus*.

## 2. Materials and Methods

### 2.1. Plant Materials

The leaves from *C. citratus* (DC) Stapf, *C. nardus* (L.) Rendle and *C. schoenanthus* (L.) Spreng were collected, early morning, from the botanical garden of the Research Institute in Applied Sciences and Technologies (IRSAT) (latitude 12°25′ N, longitude 1°29′ W), Ouagadougou (Burkina Faso). Voucher specimens were deposited at the herbarium of the Center of diversity (University Joseph KI-ZERBO, Ouagadougou) under numbers 17,940, 17,950, and 17,952 for *C. citratus*, *C. nardus*, and *C. schoenanthus*, respectively. 

### 2.2. Extraction of the Essential Oils 

A mass of 400 g of the fresh leaves from *C. citratus*, *C. nardus*, and *C. schoenanthus* were submitted to hydrodistillation for 3 h, using a Clevenger type apparatus. The recovered oils were dried over anhydrous sodium sulfate and stored in darkness before use. The yield of each oil was calculated on fresh weight basis.

### 2.3. Chemical Composition of Essential Oils

Qualitative and quantitative analysis of the EOs were performed using an Agilent 6890N gas chromatograph (Agilent 7890A, Palo Alto, CA, USA), equipped with a DB-5 capillary column (30 m × 0.25 mm, 0.25 μm stationary film thickness) and a flame ionization detector (FID) interfaced with an Agilent MS model 5975. Analytical conditions were as follows: oven temperature programmed from 60 °C to 165 °C at 8 °C/min and from 165 °C to 280 °C at 20 °C/min, with 1 min post-run at 280 °C; injection of 1 μL (1/100 in acetone) in split mode (1:150); carrier gas, helium at 1.0 mL/min; injector and detector temperature were at 250 °C and 280 °C respectively. The MS working in electron impact mode at 70 eV; electron multiplier, 1500 V; ion source temperature, 230 °C; mass spectra data were acquired in the scan mode in *m*/*z* range 33–450.

### 2.4. Fungal Species

*Aspergillus flavus* (GenBank accession number OL907105) and *Aspergillus parasiticus* (GenBank accession number OL907106) from the culture collection of the University of Western Brittany (Brest, France) were used in the present study. 

The cultures were maintained on Potato Dextrose Agar (PDA) at 25 ± 2 °C. The well-grown colonies were sub-cultured for a week before the experiments.

### 2.5. Determination of the Antifungal Activity

Contact-dependent antifungal activity of EOs by agar suspension was assessed following the procedure of Prakash et al. [19] with minor modifications. EOs were separately dissolved in dimethyl sulfoxide (DMSO) and added into molten PDA to achieve concentrations ranging between 0.25 and 5.0 µL/mL. Culture plates were prepared by pouring 20 mL PDA into sterilized Petri dishes. A 5 mm disc of the actively growing 7-day-old test fungi was placed at the center of the plate and incubated at 25 ± 2 °C for 7 days. An unexposed control set without EO was kept parallel to each treatment. Radial growth of fungus mycelium was measured in the test and control plates after 7 days. All antifungal experiments were carried out in triplicates. Growth inhibition was calculated as the percentage of inhibition of radial growth relative to the control according to a formula of Pandey et al. [20]: Percent mycelial inhibition = $\frac{dC-dT}{dC}\times 100$.

Where *dC* = average mycelial growth in control and *dT* = average mycelial growth in treatment.

#### 2.5.1. Determination of Minimum Inhibitory Concentration and Minimum Fungicidal Concentration

The broth microdilution method was used to determine the minimum inhibitory concentration (MIC) according to the Clinical and Laboratory Standards Institute (CLSI) with slight modifications [21]. EO solubility in the medium was enhanced with 10% dimethyl sulfoxide (DMSO). Serial dilutions of the EOs from 0.039 to 5 µL/mL were prepared in duplicate in a 96-well microtiter plate (96 U-shaped wells; Dynatech Laboratories, Inc., Alexandria, Va.). The final volume was set to 200 µL by adding 100 µL of the inoculum at 10^6^ spore/mL. Negative control without EO was prepared under the same conditions to assess the spore growth. An amount of 40 mL of 0.2 mg/mL p-iodonitrotetrazolium violet (INT) dissolved in water was added to the microplate wells [22]. The plates were incubated at 25 °C for 72 h under aerobic conditions. The MIC was recorded as the lowest concentration of the EO that inhibited antifungal growth. Fungal growth was determined by observing the color change of INT in the microplate wells.

To determine the minimum fungicidal concentration (MFC) of essential oil, 100 μL from the wells without visible fungus growth was subcultured on freshly prepared plates. MFC was determined as the lowest concentration of oil at which there was no revival of fungal growth occurs. Experiments were carried out in 3 replicates.

#### 2.5.2. Evaluation of the Antifungal Activity of EOs Combinations

The effect of the double combination of the EO against *A. flavus* or *A. parasiticus* was evaluated using a checkerboard assay with slight modifications [23]. Essential Oil A (EO_A_) and Essential Oil B (EO_B_) were diluted two-fold along the X-axis and the y-axis, respectively. The final volume of each well was set to 200 µL including 50 µL of appropriated diluted EOA, 50 µL of appropriated diluted EOB, and 100 µL of the inoculum 10^6^ spores /mL. The well was incubated at 25 °C for 5 days. 

The Fractional Inhibitory Concentration Index (FICI) was calculated as FIC_A_ + FIC_B_, where FIC_A_ and FIC_B_ are the minimum concentrations that inhibited fungal growth for EOs A and B, respectively. Thus, FICs were calculated as follows: FIC_A_ = (MICA combination/MIC_A_ alone) and FIC_B_ = (MIC_B_ combination/MIC_B_ alone). The results were interpreted as synergy (FIC < 0.5), addition (0.5 ≤ FIC ≤ 1), indifference (1 < FIC ≤ 4), or antagonism (FIC > 4). All experiments were done in triplicate.

### 2.6. Aflatoxin Extraction

The effect of the EO on the production of Aflatoxin B1, B2, G1, and G2 was assessed by using Petri dishes (90 mm diameter) poured with Yeast Extract Sucrose agar (YES) containing 0.5–3 µL/mL of EO alone or in combination. Then, 5 µL of a fungal suspension calibrated at 1.0 × 10^6^ spores /mL were deposited in the center of the petri dish. Petri dishes were incubated at 25 °C for 7 days. At the end of the incubation period, 8.50 g of the culture media was weighed in a sterile tube and used for aflatoxin extraction [24]. The YES was crushed in 50 mL of methanol/formic acid (25/1). The tube was centrifuged at 12,000 rpm for 20 min. An amount measuring 500 µL of the supernatant was evaporated under a nitrogen stream, and the residue was taken up in 2 mL of water-methanol (11/9) solution containing 450 mL of methanol, 119 mg of potassium bromide, and 350 µL of 4 M nitric acid. The tubes containing reconstituted extract were placed in an ice bath and were then ultrasonicated for 10 min at 40% amplitude (40% of the ultrasonicator power/frequency (130 W/20 kHz)) using continuous sonication. Sonicated solution was filtered through a PTFE filter of 0.45 μm and used for aflatoxin analysis by HPLC. 

### 2.7. Quantification of Aflatoxins by HPLC

Aflatoxins were identified by HPLC and quantified by spectrofluorescence (Shimadzu RF 20A, Japan) with the electrochemical system (Kobra Cell™ R. Biopharm Rhone Ltd., Glasgow, UK). A C-18 column (2.1 × 100 mm i.d., 3 µm particle size, Inertsil ODS-3, Japan) was used. The mobile phase was water-methanol (55/45; *v*/*v*) containing 119 mg of potassium bromide and 350 mL of nitric acid. The mobile phase isocratically delivered at 0.8 mL/min. The fluorescence detector was operated at an excitation wavelength of 362 nm and an emission wavelength of 425 nm. A five-point calibration curve was constructed using AF standards (TSL-108, Biopharm Rhone Ltd., Glasgow, UK) of 0.1–100 µg/kg. The LOD and LOQ were 0.3 µg/kg and 1 µg/kg, respectively.

### 2.8. Statistical Analysis

The data were presented as mean ± standard deviation. Analyses of variance (ANOVA) of data were carried out with XLStat PRO version 7.5.2 to compare the effects of the EOs.

## 3. Results

### 3.1. Extraction Yields of Essential Oils

The yield of EOs varied from 0.82% to 1.37% (Table 1). The highest yield was obtained with the EO from *C. nardus* and the lowest with that from *C. citratus*.

### 3.2. Chemical Composition of Essential Oils

A total of two, six, and eight compounds were identified in the EOs from *C. citratus*, *C. nardus* and *C. schoenanthus*, respectively (Table 2). Oxygenated monoterpenes were dominant compounds in the three oils, followed by hydrocarbon sesquiterpenes and hydrocarbon monoterpenes. Geranial (55.2%) and Neral (44.7%) were the main compounds of the oil from *C. citratus*. Citronellal (41.7%) and geraniol (20.8%) were predominant in the oil from *C. nardus*. The EO from *C. schoenanthus* was characterized by its high content of piperitone (59.8%).

### 3.3. Antifungal Activity of Essential Oils

#### 3.3.1. Mycelial Growth Inhibition 

Three EOs had dose-dependent effects on the growth of both *A. flavus* and *A. parasiticus*. Both *C. citratus* and *C. nardus* exhibited the highest growth inhibition effects on both *A. flavus* and *A. parasiticus*, which growth was completely inhibited at 1.5 µL/mL, whereas *C. schoenanthus* completely inhibited both strains at 2.5 µL/mL (Table 3 and Table 4).

#### 3.3.2. Antifungal Activity of Essential Oils Tested Alone on *Aspergillus flavus* and *Aspergillus parasiticus*

Both *A. flavus* and *A. parasiticus* were sensitive to the three EOs (Table 5). The MICs values varied from 1.25 to 2.50 µL/mL and from 1.25 to 2.25 µL/mL for *A. flavus* and *A. parasiticus*, respectively. *C. nardus* essential oil exhibited the highest inhibitory activity against *A. flavus*, while *C. citratus* and *C. nardus* were the most effective against *A. parasiticus*. The EO of *C. nardus* exhibited the highest fungicidal activities against both *A. flavus* and *A. parasiticus* with the MFC of 1.50 ± 0.16 and 1.75 ± 0.22 µL/mL, respectively, while the lowest activities were observed with *C. schoenanthus* with the MFC of 3.25 ± 0.33 and 2.75 ± 0.33 µL/mL for *A. flavus* and *A. parasiticus*, respectively.

#### 3.3.3. Antifungal Activity of Essential Oil Pair Combinations

The Fractional Inhibitory Concentration (FIC) indices varied from 0.14 to 0.52 and from 0.06 to 1.00 for *A. flavus* and *A. parasiticus*, respectively (Table 6). These values of the FIC indices showed additive and synergistic of both EOs against both fungi. The paired combination of *C. citratus* and *C. nardus* and that of *C. nardus* and *C. schoenanthus* exhibited synergetic effects against *A. flavus* and *A. parasiticus*, respectively. 

### 3.4. Antiaflatoxinogenic Activity of Essential Oils on the Production of Aflatoxin 

The synthesis of aflatoxin B1 was entirely inhibited by the essential oils of *C. citratus* and *C. schoenanthus* at 1 µL/mL, while that from *C. nardus* totally inhibited its production at 1.25 µL/mL. The production of aflatoxins B2 and G1 was completely inhibited at 0.50 and 0.75 µL/mL by *C. citratus* and *C. schoenanthus* EOs, respectively, against 1.25 and 1.50 µL/mL, respectively, for the essential oil of *C. nardus*. EOs from *C. citratus*, *C. schoenanthus* and *C. nardus* totally inhibited the synthesis of aflatoxins G2 at 0.50, 0.75, and 1 µL/mL, respectively (Table 7, Table 8 and Table 9). 

### 3.5. Antiaflatoxinogenic Activity of Essential Oil Pair Combinations on the Production of Aflatoxin

Only the pair combination of the EO from *C. citratus* and *C. schoenanthus* completely inhibited the production of aflatoxin B1, B2, G1, and G2. The pair combination of *C. citratus*/*C. nardus* completely inhibited the production of aflatoxin B2, G1, and G2, and reduced that from aflatoxin B1 at 99%. The combination of the EO from *C. nardus* and *C. schoenanthus* exhibited a low effect on the production of aflatoxin B1, B2, G1, and G2 (Table 10).

## 4. Discussion

### 4.1. Extraction Yield and Chemical Composition of the Essential Oils

The extraction yields of EOs obtained in the present study were in the range of 0.7–0.8%, and 0.14–1.33% previously reported for *C. citratus* and *C. nardus,* respectively [25,26,27,28,29]; whereas that of *C. schoenanthus* was lower than those found in the literature which were between 1.4% and 3% [30,31,32,33]. The previous studies showed that *C. schoenanthus* had the highest yield in EO, followed by *C. nardus* and *C. citratus,* while in the present study the highest yield was obtained with *C. nardus*, followed by *C. schoenanthus* and *C. citratus*. This could be due to the differences between species [31], in extraction techniques [30], and in environmental conditions [34].

The EOs from *C. citratus*, *C. nardus*, and *C. schoenanthus* were characterized by low numbers of components (2, 6, and 8, compared to 12, 17, and 17, respectively) reported by Sonker et al. [35], Verma et al. [34], and Bellik et al. [30], respectively.

The citral content (99.99%) of the EO oil from *C. citratus* was higher than 63–91.47%, 75–77.4%, and 58.9–92.28% reported in Cameroon and India, Benin and Brazil, Cameroon and Burkina Faso [26,27,28,29,30,31,32,33,34,35,36,37,38], respectively. The major components of the EO from *C. nardus* were different from geraniol (35.7%), trans-citral (22.7%), cis-cistral (14.2%), gernayl acetate (9.7%), citronellal (5.8%), and citronellol (4,6%) described in Thailand [39], citronellal (27.87%), β-citronellol (11.85%), neral (11.21%), geraniol (22.77%) and geranial (14.54%); geraniol (33.88%), citronellal (27.55%), and citronellol (14.40%) both found in Brazil [28] and β-citronellal (35.9%), β-citronellol (11.6%) and nerol (24.3%) reported in Benin [31].The major components of the EOs from *C. schoenanthus* were similar to those reported for the same species from Algeria, Benin, and Togo [32,33,40]. Qualitative and quantitative differences in essential oil composition could be attributed to environmental conditions, which are influenced by geographical locations [41].

### 4.2. Antifungal Activity of Essential Oils Tested Alone on Aspergillus flavus and Aspergillus parasiticus

The present study shows that the EOs from *C. citratus*, *C. nardus*, and *C. schoenanthus* exerted antifungal activities against *A. flavus* and *A. parasiticus*. These findings were consistent with the previous studies, which reported the antifungal activities of the three EOs against *A. flavus*, *A. fumigatus*, *A. niger*, *A. ochraceus*, and *A. westerdijkiae* [30,42,43]. In the present study, the *C. nardus* EO exhibited the highest fungicidal activities against both *A. flavus* and *A. parasiticus,* followed by *C. citratus* and by *C. schoenanthus*. It has been reported that the functional groups of the major components of essential oils play an important role in their antifungal activity [44]. The highest fungicidal activity from *C. nardus* could be ascribed to the presence of citronellal and geraniol as main components [43], whereas that from *C. citratus* could be related to its two main components, neral and geranial [45]. Caärdenas-Ortega et al. [46] attributed to piperitone the fungicidal activity of the EO of *Chrysactinia mexicana* against *A. flavus*. Kalemba and Kunicka [47] showed that the antifungal activity of essential oils according to their major components followed the rule phenols > aldehydes > ketones > alcohols > esters > hydrocarbons. According to this hypothesis, the EO from *C. citratus* should exert the highest fungicidal against both *A. flavus* and *A. parasiticus* followed by *C. schoenanthus* and *C. nardus*, but the opposite results were obtained. This could be due to the presence of the minor components and the interaction between EO components [48]. The interaction between the components of an EO could lead to a synergistic or antagonistic effect, which could increase or decrease its activity [49]. The antifungal activities of the essential oil components have been attributed to several mechanisms, including the leakage of intracellular biological macromolecules, the inhibition of ATPase activity, the intracellular generation of reactive oxygen species (ROS), the destruction of the cytoplasmic membrane, and the damage of mitochondria and DNA [50,51]. 

### 4.3. Antifungal Activity of Essential Oils Tested in Combination on Aspergillus flavus and Aspergillus parasiticus

No antagonistic effect occurred when the three EOs were pair combined. Only additive and synergistic effects were observed. These results can be explained by the presence of the specific component into individual EO and by their dose-dependent interaction [52]. In this study, only combination with *C. nardus* exhibited a synergistic effect against both *A. flavus* and *A. parasiticus*, indicating a specific synergistic interaction between the components of the EO of *C. nardus* and those of *C. citratus* and *C. schoenanthus*. Tang et al. [53] observed a synergistic effect of the citral and geraniol against *Aspergillus spp*. The mechanism of the antifungal synergistic effect remains unclear. The same author reported that the citral exerted its antifungal activity against *A. flavus* and *A. ochraceus* mainly by downregulating the sporulation- and growth-related genes, whereas geraniol acted by inducing the intracellular ROS accumulation. 

### 4.4. Antiaflatoxinogenic Activity of Essential Oils Tested Alone or in Combination

In this study, the synthesis of aflatoxins B1 was entirely inhibited by the Eos of *C. citratus*, *C. nardus*, and *C. schoenanthus* at 1 μL/mL. This concentration is higher than those obtained with *C. citratus* by Paranagama et al. [54], Singh et al. [55], and Sonker et al. [35], which were, respectively, 0.2, 0.5, and 0.8 μL/mL. The EO of *C. nardus* at 0.3 μL/ mL inhibited the production of aflatoxin at 61.5% [56], and completely inhibited their synthesis at 0.6 mg/mL [57]. We did not find any previous studies on the EO of *C. schoenanthus* on aflatoxins, but its major component, piperitone, has been reported to inhibit 3-acetyldeoxynivalenol production by *Fusarium graminearum* [58]. Previous studies showed that citral was able to modulate the downregulation of mycotoxin biosynthetic genes and Z-citral completely inhibited aflatoxin B1 at 1.0 µL/mL [59,60]. Authors showed that antioxidants (such as citral, cinnamaldehyde, and eugenol) can significantly reduce aflatoxin production, whereas oxidants enhance aflatoxin productions [61,62]. Bioactive plant compounds like carvacrol, cinnamaldehyde, eugenol, limonene, terpineol, thymol, and turmerone are reported to be effective in suppressing aflatoxin productions [63,64].

The effect of combinations on the synthesis of aflatoxins could be due to the synergy between different components of EOs. According to Chandra [56], *C. nardus* and *C. caseius* EOs in combination caused synergistic inhibition of aflatoxin production as compared to their individual oils. The similarity of the chemical compounds of two EOs from plants of the same genus could lead to a synergistic activity on one or more targets of molds and prevent the synthesis of toxins if they are used in combination [19,59,65]. The antiaflatoxin actions of plant EOs may be related to inhibition of aflatoxin biosynthesis involving lipid peroxidation and oxygenation [19]. Therefore, EOs may contain some inhibitor substances that interfere with some steps in the metabolic pathways that control the biosynthesis of aflatoxin B1 in both Aspergillus strains [66].

## 5. Conclusions

The results of the present study showed that the EOs from *C. citratus*, *C. nardus*, and *C. schoenanthus* exerted good antifungal and antiaflatoxinogenic effects against both *A. flavus* and *A. parasiticus*, and these effects were enhanced when combined. The effects were dose-dependent on fungal growth and aflatoxin production. The study also showed that the combination of essential oils from *C. citratus*, *C. nardus*, and *C. schoenanthus* produced synergistic and additive effects. However, in vivo studies should be considered to exploit the activity of these essential oils in the post-harvest protection of foodstuffs against fungal contamination and the production of aflatoxins.

## Figures and Tables

**Table 1:** Extraction yields of essential oils from three aromatic plants.

Plants	Yield % (*w*/*w*)
*Cymbopogon citratus*	0.82 ± 0.14
*Cymbopogon nardus*	1.37 ± 0.18
*Cymbopogon schoenanthus*	0.95 ± 0.15

**Table 2:** Chemical composition of analyzed essential oil samples.

Compounds	Retention Index	*Cymbopogon citratus* (%)	*Cymbopogon* *nardus* (%)	*Cymbopogon* *schoenanthus* (%)
2-carene	999	-	-	16.4
Limonene	1028	-	-	1.8
Citronellal	1158	-	41.7	-
Citronellol	1230	-	8.0	-
Neral	1242	44.7	-	-
Piperitone	1252	-	-	59.8
Geraniol	1253	-	20.8	-
Geranial	1268	55.2	-	-
β-Elemene	1372	-	11.0	3.4
α−Copaene	1390	-	3.7	-
β-Caryophyllene	1415	-		3.1
Β-himachalene	1499	-		1.4
Hedycaryol	1520	-	7.4	
Elemol	1545	-	-	8.5
β-eudesmol	1650	-		3.7
Hydrocarbon monoterpenes		-	-	18.2
Oxygenated monoterpenes		99.9	77.9	72
Hydrocarbon sesquiterpenes		-	14.7	7.9
Total		99.9	92.6	98.1

**Table 3:** Inhibition of mycelial growth of *Aspergillus flavus* (%), depending on the concentration of the essential oil.

Concentration of EOs (µL/mL)	Essential Oils		
	*Cymbopogon citratus*	*Cymbopogon nardus*	*Cymbopogon schoenanthus*
0.5	55.4 ± 2.1 ^a^	48.8 ± 1.4 ^a^	24 ± 1.0 ^a^
1	87 ± 1.1 ^b^	79.9 ± 1.6 ^b^	51.1 ± 2.7 ^b^
1.5	100 ± 0.0 ^c^	100 ± 0.0 ^c^	65.3 ± 1.7 ^c^
2	100 ± 0.0 ^c^	100 ± 0.0 ^c^	88.5 ± 1.5 ^d^
2.5	100 ± 0.0 ^c^	100 ± 0.0 ^c^	100 ± 0.0 ^e^

Values are means (*n* = 3) ± SD. Letters a to e are comparison indices. The means followed by the same letter in the same column are not significantly different according to ANOVA multiple comparison tests (*p* < 0.05).

**Table 4:** Inhibition of mycelial growth of *Aspergillus parasiticus* (%), depending on the concentration of the essential oil.

Concentration of EOs (µL/mL)	Essential Oils		
	*Cymbopogon citratus*	*Cymbopogon nardus*	*Cymbopogon schoenanthus*
0.5	59.1 ± 1.8 ^a^	54.2 ± 0.9 ^a^	34.5 ± 1.6 ^a^
1	85.7 ± 1.8 ^b^	87.7 ± 1.1 ^b^	53.2 ± 1.3 ^b^
1.5	100 ± 0.0 ^c^	100 ± 0.0 ^c^	71.9 ± 1.46 ^c^
2	100 ± 0.0 ^c^	100 ± 0.0 ^c^	89.1 ± 1.3 ^d^
2.5	100 ± 0.0 ^c^	100 ± 0.0 ^c^	100 ± 0.0 ^e^

Values are means (*n* = 3) ± SD. Letters a to e are comparison indices. The means followed by the same letter in the same column are not significantly different according to ANOVA multiple comparison tests (*p* < 0.05).

**Table 5:** Minimum inhibitory concentrations and minimum fungicidal concentrations of essential oils.

EOs	MIC (µL/mL)		MFC (µL/mL)	
	*A. flavus*	*A. parasiticus*	*A. flavus*	*A. parasiticus*
*Cymbopogon citratus*	1.50 ± 0.12 ^a^	1.25 ± 0.08 ^a^	2.0 ± 0.22 ^a^	2.33 ± 0.11 ^ab^
*Cymbopogon nardus*	1.25 ± 0.09 ^a^	1.25 ± 0.03 ^a^	1.50 ± 0.16 ^a^	1.75 ± 0.22 ^a^
*Cymbopogon schoenanthus*	2.50 ± 0.12 ^b^	2.25 ± 0.08 ^b^	3.25 ± 0.33 ^b^	2.75 ± 0.33 ^b^

Values are means (*n* = 3) ± SD. Letters a to b are comparison indices. The means followed by the same letter in the same column are not significantly different according to ANOVA multiple comparison tests (*p* < 0.05).

**Table 6:** Effect of essential oil combinations on *Aspergillus flavus* and *Aspergillus parasiticus*.

Combinations of EOs	MIC in Combination (µL/mL)	FIC Value		Interaction	
		A. flavus *	*A. parasiticus ^+^*	*A. flavus*	*A. parasiticus*
*C. citratus/C. nardus*	0.31/0.63	0.14	0.75	Synergistic	Additivity
*C. citratus/C. schoenanthus*	0.63/1.13	0.52	1.00	Additivity	Additivity
*C. nardus/C. schoenanthus*	0.04/0.07	0.52	0.06	Additivity	Synergistic

* *Aspergillus flavus* (UBOCC-A-106031) ^+^ *Aspergillus parasiticus* (UBOCC-A-111042).

**Table 7:** Effect of *C. citratus* essential oil on aflatoxin production (µg/kg) after 7 days exposure to different concentrations.

Essential Oils	EO Concentrations (µL/mL)	AFB1	AFB2	AFG1	AFG2
	0	1620.7 ± 44.5 ^c^	74.4 ± 3.7 ^b^	1341.7 ± 49 ^b^	168.6 ± 37 ^b^
*Cymbopogon* *citratus*	0.50	144.3 ± 9.4 ^b^	0.0 ± 0.0 ^a^	43.7 ± 4.3 ^a^	0.0 ± 0.0 ^a^
	0.75	103.8± 11.2 ^b^	0.0 ± 0.0 ^a^	0.0 ± 0.0 ^a^	0.0 ± 0.0 ^a^
	1.00	0.0 ± 0.0 ^a^	0.0 ± 0.0 ^a^	0.0 ± 0.0 ^a^	0.0 ± 0.0 ^a^
	1.25	0.0 ± 0.0 ^a^	0.0 ± 0.0 ^a^	0.0 ± 0.0 ^a^	0.0 ± 0.0 ^a^
	1.50	0.0 ± 0.0 ^a^	0.0 ± 0.0 ^a^	0.0 ± 0.0 ^a^	0.0 ± 0.0 ^a^
	1.75	0.0 ± 0.0 ^a^	0.0 ± 0.0 ^a^	0.0 ± 0.0 ^a^	0.0 ± 0.0 ^a^
	2.00	0.0 ± 0.0 ^a^	0.0 ± 0.0 ^a^	0.0 ± 0.0 ^a^	0.0 ± 0.0 ^a^
	2.25	0.0 ± 0.0 ^a^	0.0 ± 0.0 ^a^	0.0 ± 0.0 ^a^	0.0 ± 0.0 ^a^
	2.50	0.0 ± 0.0 ^a^	0.0 ± 0.0 ^a^	0.0 ± 0.0 ^a^	0.0 ± 0.0 ^a^

Values are means (*n* = 3) ± SD. Letters a to c are comparison indices. The means followed by the same letter in the same column are not significantly different according to ANOVA multiple comparison tests (*p* < 0.05).

**Table 8:** Effect of *C. nardus* essential oil on aflatoxin production (µg/kg) after 7 days exposure to different concentrations.

Essential Oil	EO Concentrations (µL/mL)	AFB1	AFB2	AFG1	AFG2
	0	1620.7 ± 44.5 ^d^	74.4 ± 3.7 ^d^	1341.7 ± 49 ^e^	168.6 ± 37.^d^
*Cymbopogon nardus*	0.50	690.9 ± 10.2 ^c^	66.8 ± 2.5 ^c^	1314.07 ± 49.7 ^e^	70.8 ± 3.4 ^c^
	0.75	552.6 ± 38.22 ^b^	43.2 ± 3.^b^	970.9 ± 50.2 ^d^	35.4 ± 3.0 ^b^
	1.00	30.9 ± 4.3 ^a^	45.9 ± 2.4 ^b^	778.7 ± 7.6 ^c^	0.0 ± 0.0 ^a^
	1.25	0.0 ± 0.0 ^a^	0.0 ± 0.0 ^a^	361.1± 7.6 ^b^	0.0 ± 0.0 ^a^
	1.50	0.0 ± 0.0 ^a^	0.0 ± 0.0 ^a^	0.0 ^a^	0.0 ± 0.0 ^a^
	1.75	0.0 ± 0.0 ^a^	0.0 ± 0.0 ^a^	0.0 ^a^	0.0 ± 0.0 ^a^
	2.00	0.0 ± 0.0 ^a^	0.0 ± 0.0 ^a^	0.0 ^a^	0.0 ± 0.0 ^a^
	2.25	0.0 ± 0.0 ^a^	0.0 ± 0.0 ^a^	0.0 ^a^	0.0 ± 0.0 ^a^
	2.50	0.0 ± 0.0 ^a^	0.0 ± 0.0 ^a^	0.0 ^a^	0.0 ± 0.0 ^a^

Values are means (*n* = 3) ± SD. Letters a to e are comparison indices. The means followed by the same letter in the same column are not significantly different according to ANOVA multiple comparison tests (*p* < 0.05).

**Table 9:** Effect of *C. schoenanthus* oil on aflatoxin production (µg/kg) after 7 days exposure to different concentrations.

Essential Oil	EO Concentrations (µL/mL)	AFB1	AFB2	AFG1	AFG2
	0	1620.7 ± 44.5 ^c^	74.4 ± 3.7 ^b^	1341.7 ± 49 ^c^	168.6 ± 37 ^c^
*Cymbopogon schoenanthus*	0.50	196 ± 9.9 ^b^	14.1 ± 0.7 ^a^	650.7 ± 42.1 ^b^	19.2 ± 3.2 ^b^
	0.75	51.4 ± 2.5 ^a^	0.0 ± 0.0 ^a^	0.0 ± 0.0 ^a^	0.0 ± 0.0 ^a^
	1.00	0.0 ± 0.0 ^a^	0.0 ± 0.0 ^a^	0.0 ± 0.0 ^a^	0.0 ± 0.0 ^a^
	1.25	0.0 ± 0.0 ^a^	0.0 ± 0.0 ^a^	0.0 ± 0.0 ^a^	0.0 ± 0.0 ^a^
	1.50	0.0 ± 0.0 ^a^	0.0 ± 0.0 ^a^	0.0 ± 0.0 ^a^	0.0 ± 0.0 ^a^
	1.75	0.0 ± 0.0 ^a^	0.0 ± 0.0 ^a^	0.0 ± 0.0 ^a^	0.0 ± 0.0 ^a^
	2.00	0.0 ± 0.0 ^a^	0.0 ± 0.0 ^a^	0.0 ± 0.0 ^a^	0.0 ± 0.0 ^a^
	2.25	0.0 ± 0.0 ^a^	0.0 ± 0.0 ^a^	0.0 ± 0.0 ^a^	0.0 ± 0.0 ^a^
	2.50	0.0 ± 0.0 ^a^	0.0 ± 0.0 ^a^	0.0 ± 0.0 ^a^	0.0 ± 0.0 ^a^

Values are means (*n* = 3) ± SD. Letters a to c are comparison indices. The means followed by the same letter in the same column are not significantly different according to ANOVA multiple comparison tests (*p* < 0.05).

**Table 10:** Effect of essential oil pair combinations on the production of aflatoxin B1, B2, G1, and G2 (µg/kg).

Combinations of EOs	MIC in Combination	AFB1	AFB2	AFG1	AFG2
Control	-	1626.5 ± 13.9	30.1 ± 3.0	890.2 ± 11.7	32.6 ± 2.2
*C. citratus*/*C. nardus*	0.31/0.63	8.7 ± 0.03	0.0	0.0	0.0
*C. citratus*/*C. schoenanthus*	0.63/1.13	0.0	0.0	0.0	0.0
*C. nardus*/*C. schoenanthus*	0.04/0.07	1475.0 ± 4.7	17.1 ± 4.3	756.0 ± 3.2	30.6 ± 0.8

Values are mean (*n* = 3) ± SD.

## Data Availability

Data are contained within the article.
